# Supplementary material for: In Vitro Human Metabolism and Inhibition Potency of Verbascoside for CYP Enzymes
Source: Molecules. 2019 Jun 11;24(11):2191. doi: 10.3390/molecules24112191 (PMC6600574; doi:10.3390/molecules24112191)
Supplement: Supplementary file 1 [file molecules-24-02191-s001.pdf]

Supplementary Material

In vitro human metabolism and inhibition potency of verbascoside for CYP enzymes

Anna-Mari Reid [1], Risto Juvonen [2], Pasi Huuskonen [2], Marko Lehtonen [3], Markku Pasanen [2] and Namrita Lall [2, *]

[1] Department of Plant and Soil Sciences, University of Pretoria, Pretoria, South Africa; annamarikok@gmail.com
[2] School of Pharmacy, Faculty of Health Sciences, University of Eastern Finland, Kuopio, Finland; risto.juvonen@uef.fi
[3] LC-MS Metabolomics Center, Biocentre, Kuopio, Kuopio, Finland; marko.lehtonen@uef.fi.
* Correspondence: namrita.lall@up.ac.za

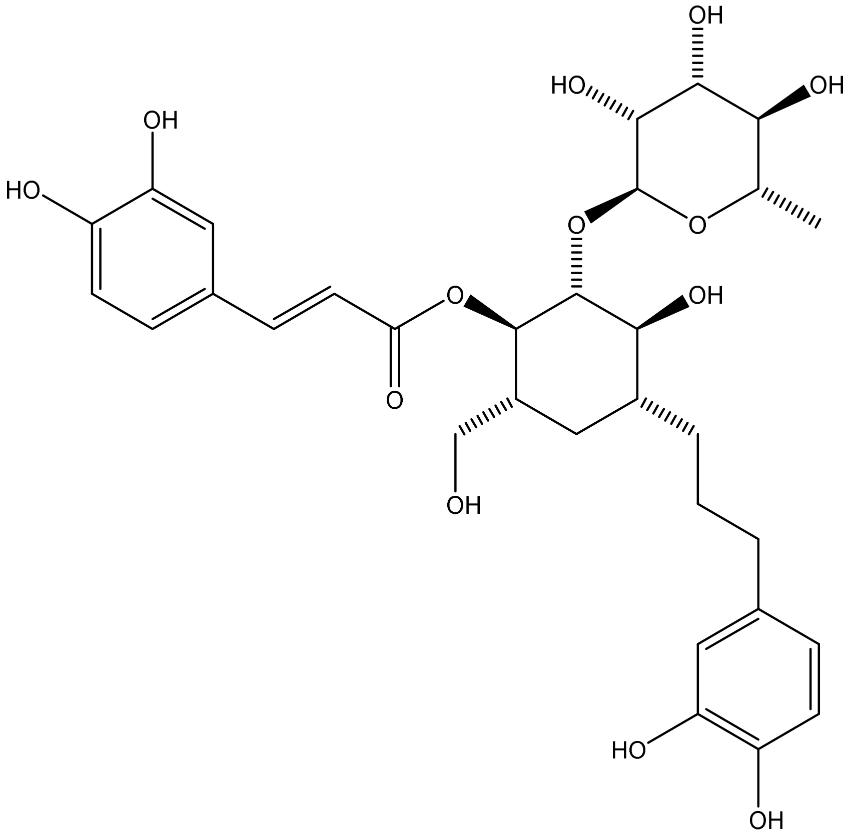

Fig 1S: Verbascoside structure

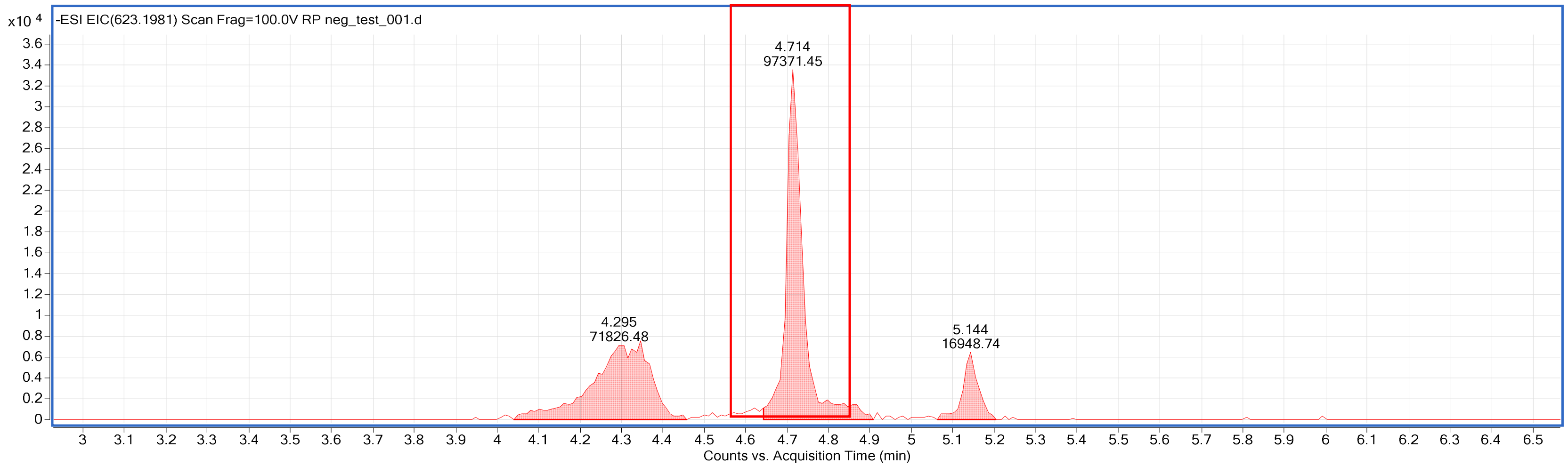

Fig 2S: The ESI EIC scan of verbascoside (Standard). The area of interest is circled in red (peak= 4.714)

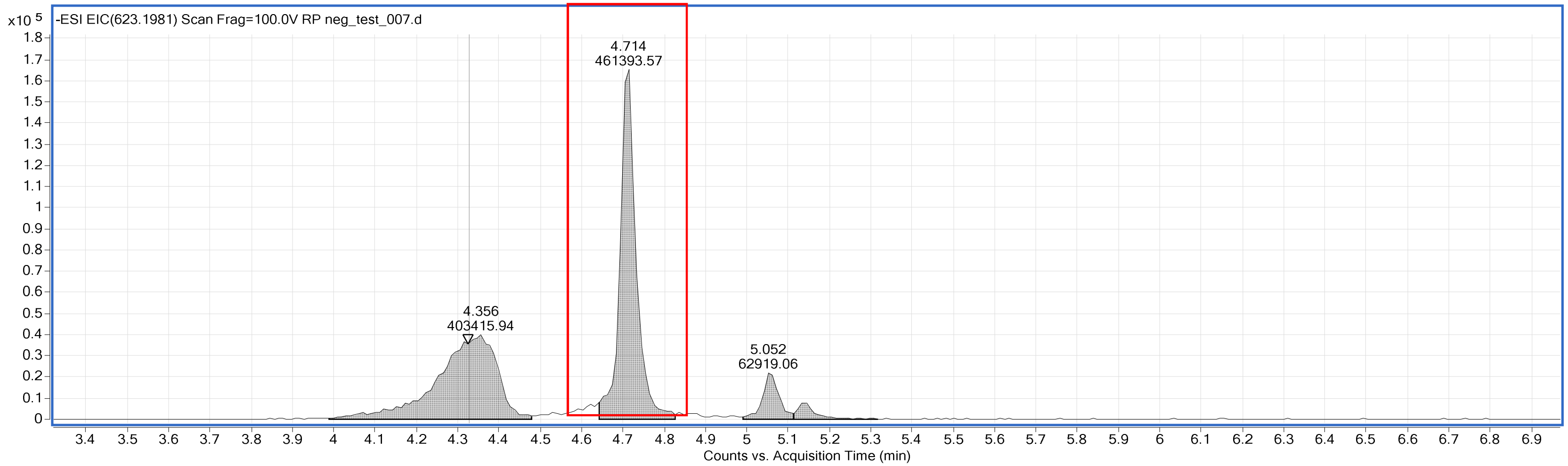

Fig 3S: The ESI EIC scan of the ethanolic extract of *Lippia scaberrima*, confirming the presence of verbascoside within the extract. The area of interest is circled in red (peak= 4.714)

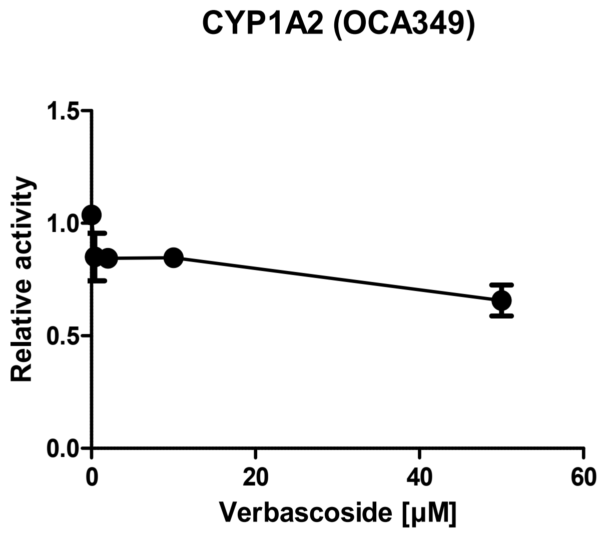

Fig 4S: The recombinant CYP 1A2 inhibition potential of verbascoside after incubation at various concentrations

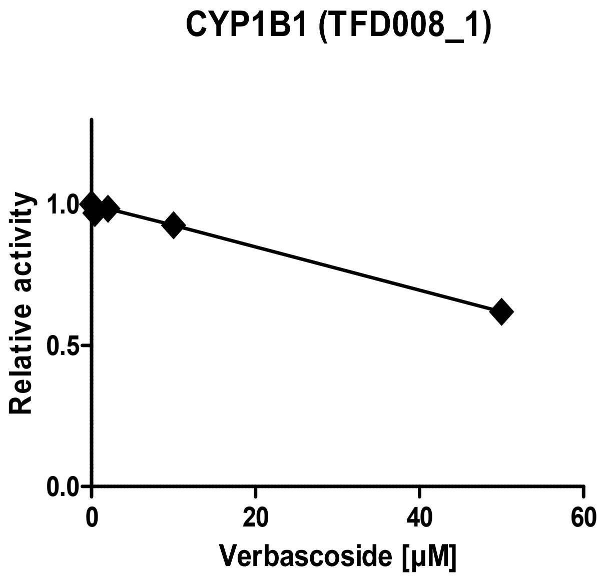

Fig 5S: The recombinant CYP 1B1 inhibition potential of verbascoside after incubation at various concentrations

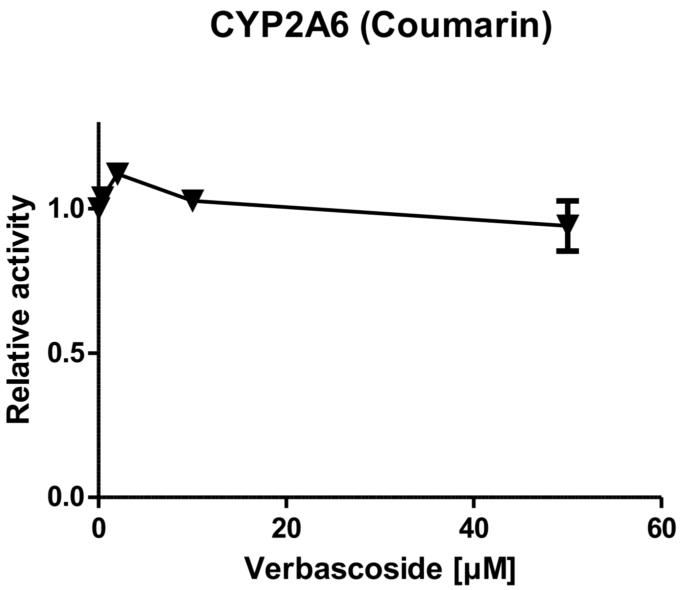

Fig 6S: The recombinant CYP 2A6 inhibition potential of verbascoside after incubation at various concentrations

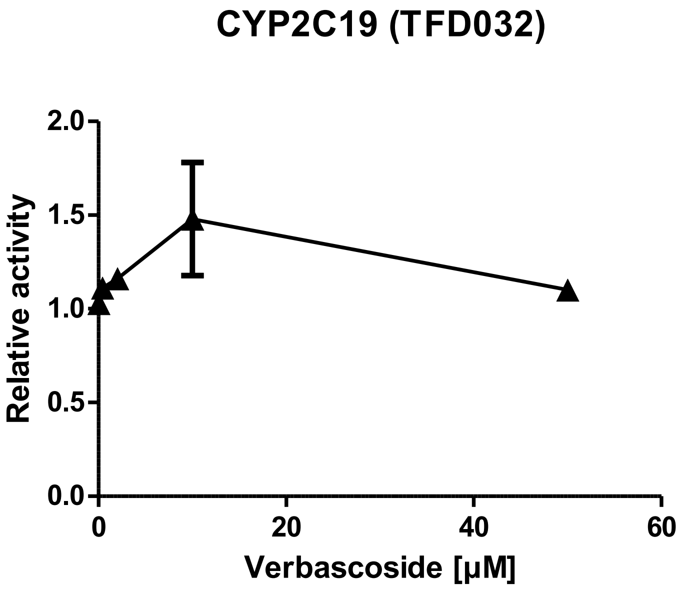

Fig 7S: The recombinant CYP 2C19 inhibition potential of verbascoside after incubation at various concentrations

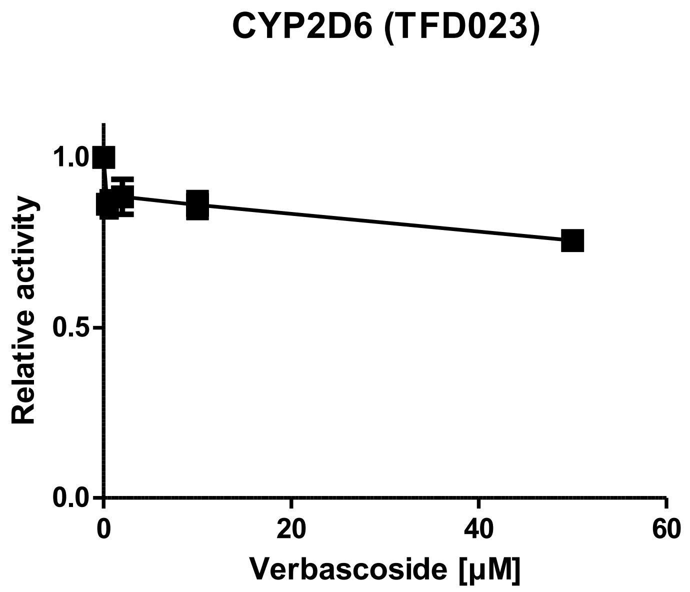

Fig 8S: The recombinant CYP 2D6 inhibition potential of verbascoside after incubation at various concentrations

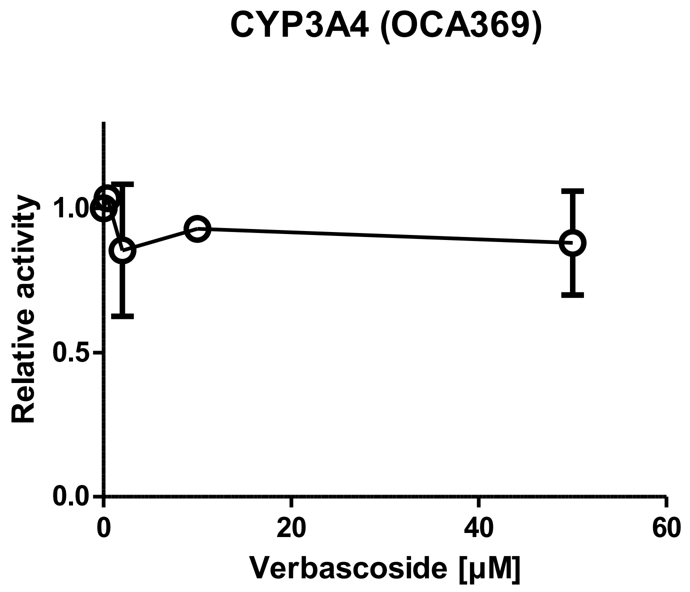

Fig 9S: The recombinant CYP 3A4 inhibition potential of verbascoside after incubation at various concentrations

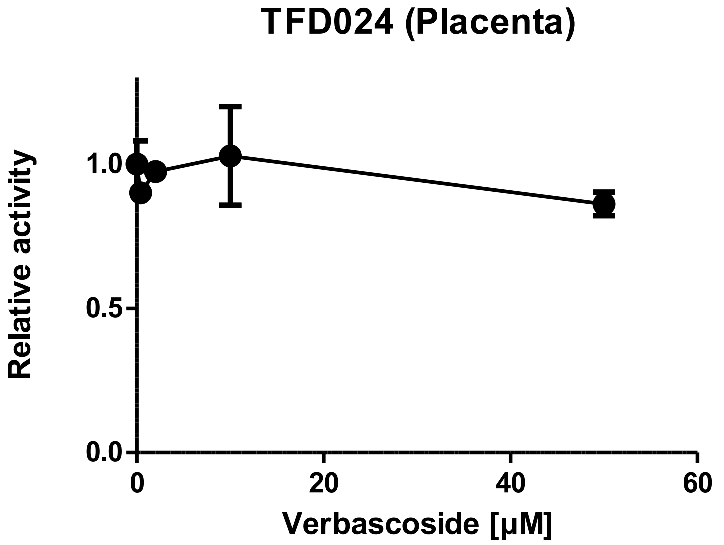

Fig 10S: The microsomal CYP1A1 inhibition potential of verbascoside after incubation at various concentrations

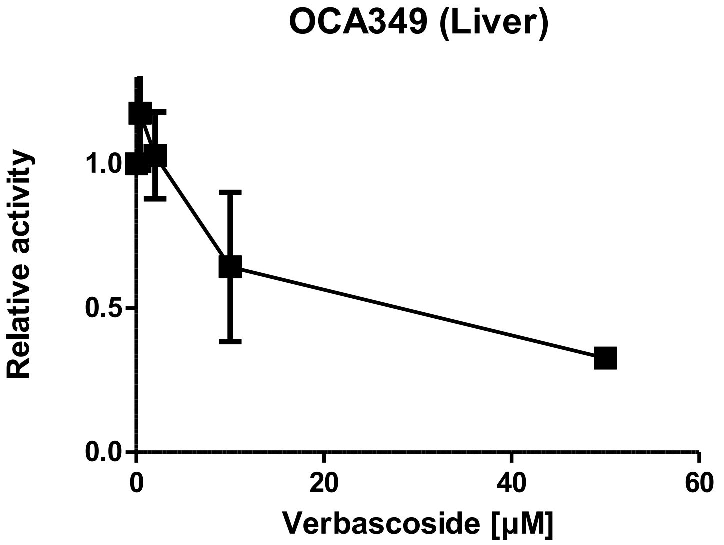

Fig 11S: The microsomal CYP1A2 inhibition potential of verbascoside after incubation at various concentrations

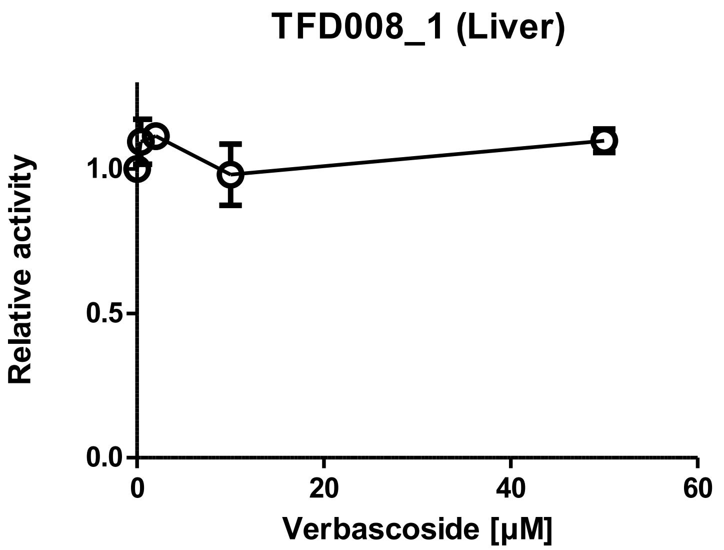

Fig 12S: The microsomal CYP1B1 inhibition potential of verbascoside after incubation at various concentrations

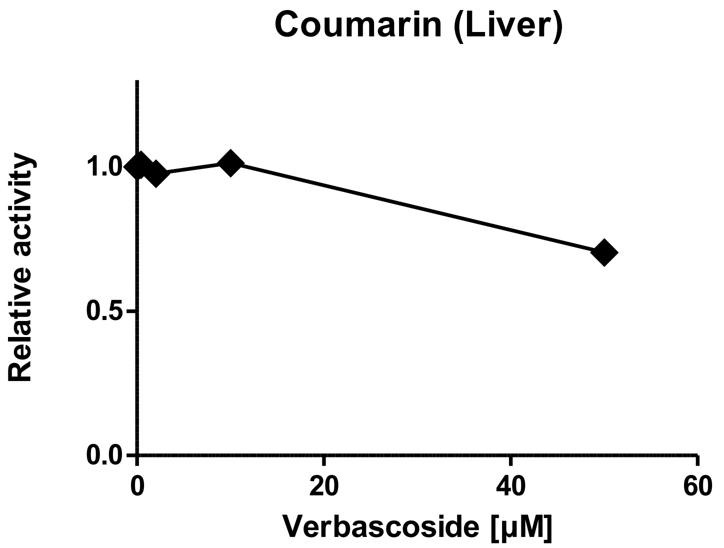

Fig 13S: The microsomal CYP2A6 inhibition potential of verbascoside after incubation at various concentrations

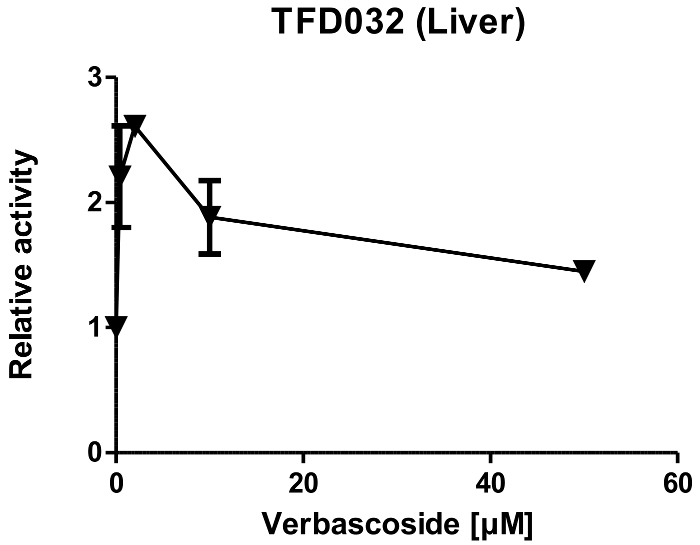

Fig 14S: The microsomal CYP2C19 inhibition potential of verbascoside after incubation at various concentrations

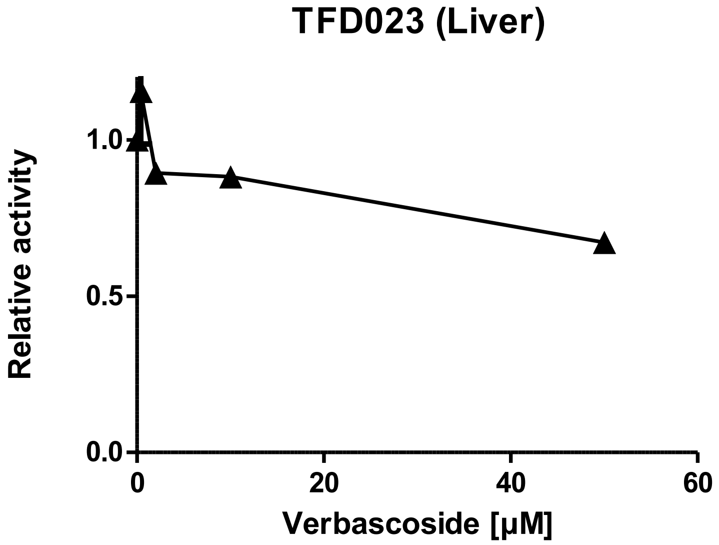

Fig 15S: The microsomal CYP2D6 inhibition potential of verbascoside after incubation at various concentrations

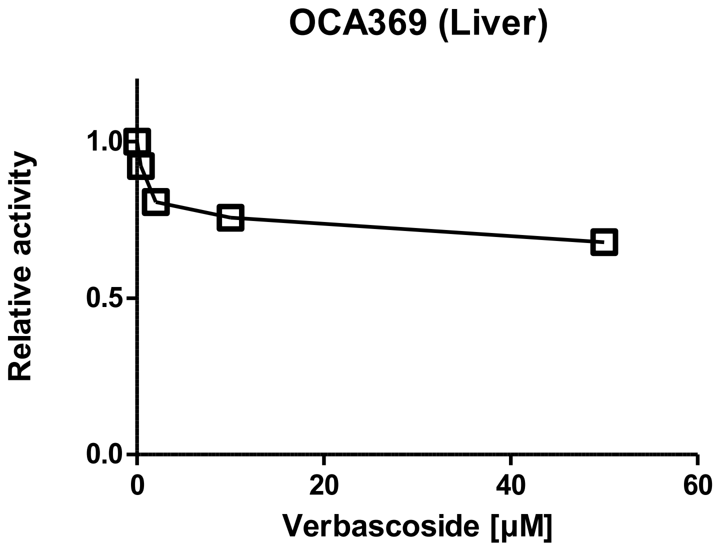

Fig 16S: The microsomal CYP3A4 inhibition potential of verbascoside after incubation at various concentrations
